# Incidence of acute otitis media from 2003 to 2019 in children ≤ 17 years in England

**DOI:** 10.1186/s12889-023-14982-8

**Published:** 2023-01-30

**Authors:** Salini Mohanty, Bélène Podmore, Ana Cuñado Moral, Thomas Weiss, Ian Matthews, Eric Sarpong, Ignacio Méndez, Nawab Qizilbash

**Affiliations:** 1 Merck & Co., Inc, Center for Observational and Real-World Evidence (CORE), Rahway, NJ USA; 2 OXON Epidemiology Ltd, Epidemiology & Statistics, Madrid, Spain; 3 MSD (UK) Ltd, Value, Access and Devolved nations (VAD), London, UK; 4 Merck & Co., Inc., Real-world Data Analytics and Innovation (RDAI), Rahway, NJ USA

**Keywords:** Acute otitis media, Recurrent AOM, Pneumococcal conjugate vaccine, United Kingdom

## Abstract

**Background:**

The 7-valent pneumococcal conjugate vaccine (PCV7) was introduced in 2006 and the 13-valent pneumococcal conjugate vaccine (PCV13) in 2010 in the UK. PCVs are active immunization for the prevention of invasive disease, pneumonia and acute otitis media (AOM) caused by *Streptococcus pneumoniae* in children.

The aim of this observational study was to estimate incidence rates (IRs) of AOM in children ≤17 years from 2003 to 2019 in England, before and after the introduction of pneumococcal conjugate vaccines (PCVs).

**Methods:**

AOM episodes were identified using Read diagnosis codes in children aged ≤17 years in the Clinical Practice Research Datalink (CPRD) Gold database from 2003 to 2019. Annual IRs with 95% confidence intervals (CI) by age group were calculated as the number of episodes/person-years (PY) at risk. Interrupted time series analyses were conducted to estimate incidence rate ratios (IRR) across post-PCV7 (2007–2009), early post-PCV13 (2011–2014) and late post-PCV13 (2015–2019) periods compared to the pre-PCV7 period (2003–2005) using generalized linear models.

**Results:**

From 2003 to 2019, 274,008 all-cause AOM episodes were identified in 1,500,686 children. The overall AOM IR was 3690.9 (95% CI 3677.1-3704.8) per 100,000 PY. AOM IRs were highest in children aged < 5 years and decreased by age; < 2 years: 8286.7 (95% CI 8216.8-8357.1); 2–4 years: 7951.8 (95% CI 7902.5-8001.4); 5–17 years: 2184.4 (95% CI 2172.1–2196.8) (per 100,000 PY). Overall AOM IRs declined by 40.3% between the pre-PCV7 period and the late-PCV13 period from 4451.9 (95% CI 4418.1-4485.9) to 2658.5 (95% CI 2628.6-2688.7) per 100,000 PY, and across all age groups. IRRs indicated a significant decrease in AOM IRs in all the post-vaccination periods, compared to the pre-PCV7 period: post-PCV7 0.87 (95% CI 0.85–0.89), early post-PCV13 0.88 (95% CI 0.86–0.91), and late post-PCV13 0.75 (95% CI 0.73–0.78).

**Conclusions:**

The AOM IRs declined during the 2003–2019 period; however, the clinical burden of AOM remains substantial among children ≤17 years in England.

**Supplementary Information:**

The online version contains supplementary material available at 10.1186/s12889-023-14982-8.

## Background

Otitis media (OM) is an inflammation of the middle ear that is associated with fever, ear pain or effusion [1–3]. It is a spectrum of diseases that includes acute otitis media (AOM), chronic suppurative otitis media (CSOM), and otitis media with effusion (OME) [1, 4]. A diagnosis of AOM requires a history of acute onset of signs and symptoms, the presence of middle ear effusion, and middle-ear inflammation [5]. Previous estimates report a global OM incidence rate of 10.85% i.e. 709 million cases each year with 51% of these occurring in children under 5 years of age [6].

OM is mainly an infectious disease, resulting from an interplay between microbial load (viral and bacterial) and immune response. Factors known to cause OM are related to these two core elements: host factors, such as age, genetic predisposition, and atopy relate to the impaired immune system, whereas environmental factors such as having siblings (generally older), group day care, and season of year are related to microbial load [7]. Other environmental factors such as environmental pollution and low socioeconomic status have also shown to be high risk factors for AOM [8].

A systematic review performed by Ngo CC., et al. confirmed that *Streptococcus pneumoniae (S. pneumoniae)* and *Haemophilus influenzae* remained the predominant bacterial pathogens, with *S. pneumoniae* the predominant bacterium in the majority reports from AOM patients [9]. In addition, the percentage of AOM caused by *S. pneumoniae* in children ranged from 8.9 to 43% in Spain [10, 11].

In the UK, antibiotic prescribing rates in children with AOM are high. A recent systematic review analyzing the use of antibiotics in ambulatory care for acutely ill children in high-income countries, including the UK, reported the antibiotic prescribing rate for AOM was 85.6% (95% confidence interval, CI 73.3–92.9%) [12]. As a result, the National Institute for Health and Care Excellence (NICE) guideline for AOM antimicrobial prescribing was updated in March 2022 to aim to limit antibiotic use and reduce antimicrobial resistance [13].

Regarding active immunization, in September 2006, the UK introduced the 7-valent pneumococcal conjugate vaccine (PCV7) into the routine childhood immunization program for children aged 2, 4, and 12–13 months, alongside a 12-month catch-up program for children younger than 2 years. In April 2010, the 13-valent PCV (PCV13) replaced the PCV7, providing additional protection against six further serotypes (1, 3, 5, 6A, 7F and 19A), and administered at 2 and 4 months of age, with a booster at 12–13 months of age [14–16]. As a result of current low rates of PCV-13 type pneumococcal disease, the UK Joint Committee on Vaccination and Immunisation (JCVI) agreed that one dose of the vaccine plus a booster should continue to provide good protection for children and the community, switching from a 2 + 1 to a 1 + 1 schedule. Hence, babies born in the UK after 1 January 2020, receive the PCV at 3 months of age, followed by a booster at 12–13 months [15, 17, 18]. In England, PCV coverage at 12 months was 90.3% in 2019–20 and increased up to 93.8% in 2021–22. PCV coverage data at 12 months was unavailable in 2020–21 due to a change in the schedule for this vaccine [19].

Since the introduction of PCVs in Europe, the incidence of both all-cause and pneumococcal AOM has declined [3, 20–23]. A recent systematic review analyzed 48 observational studies in high-income countries including the UK evaluating PCV7, PCV10 and PCV13 vaccine effectiveness (VE) in preventing all-cause OM and AOM in children aged < 5 years. In children aged < 5 years old, VE ranged between 13.2 and 39% for PCV7, and 39 to 41% for PCV13 [24]. A significant effect of pneumococcal vaccination in decreasing OM or AOM in children < 5 years old was observed, supporting the introduction of PCVs in national immunization programs. Two studies included in this systematic review were conducted in the UK. Lau WCY., et al. (2015) showed that the introduction of PCV7 and PCV13 was associated with significant decline in OM rates in the 2002–2012 period in children aged less than 10 years using primary care records [2]. Similarly, Thorrington D., et al. (2018) using hospital admissions data, showed a reduction of OM incidence in the 2004–2015 period in children under 15 years old [25]. Recent studies are needed in the UK and globally to determine whether trends in AOM incidence rates (IRs) have persisted in the late post-PCV13 years.

Higher-valent PCVs are now in late-stage clinical development for pediatric use, including a 15-valent PCV (PCV15) recently approved for use in adults and children in Europe, the US and Canada [26–28]. In Europe, PCV15 is indicated for active immunization for the prevention of invasive disease, pneumonia and AOM caused by *Streptococcus pneumoniae* in infants, children and adolescents from 6 weeks to < 18 years of age, and in individuals ≥18 years of age, PCV15 is indicated for active immunisation for the prevention of invasive disease and pneumonia caused by *Streptococcus pneumoniae* [26]. A 20-valent PCV (PCV20) is currently approved for use in adults in Europe and the US [29, 30]. To better describe the potential benefit of new higher-valent PCVs in England, it is important to understand the clinical burden of AOM. This observational study estimated IRs for all-cause AOM IRs, simple AOM and recurrent AOM, and IRs of AOM from 2003 to 2019 in children ≤17 years old in England, before and after the introduction of pneumococcal conjugate vaccines (PCVs).

## Methods

### Study design

This was a retrospective observational cohort study of children aged ≤17 years in England from 1 January 2003 to 31 December 2019 using data from the Clinical Practice Research Datalink (CPRD)-Gold database [31]. To be eligible for inclusion, children aged 1–17 years needed to have at least 12 months of medical up-to standard practice data (UTS) (measure of data quality as defined in CPRD [31]) between last practice data collection date (last data upload from each practice [31]), and study inclusion date to ensure their medical history could be assessed with continuous follow-up in the last 6 months. Children aged ≤1 year did not need to meet these eligibility criteria. Continuous follow-up was determined from the current registration date and only for patients with no interruptions to their follow-up or single interruptions ≤7 days. All children were followed from the earliest of the following events: the start of study period (1 January 2003), the date of birth, or start of data collection. The end of follow-up was the earliest of the following events: the end of the study period (31 December 2019), the end of the year in which the patient turns 17 years, death, transfer out of the practice, or end of data collection.

### Data source

The CPRD-Gold database is an ongoing primary care database of anonymized medical records from general practitioners (GP) in England. GPs are the gatekeepers of primary care and specialist referrals in the UK. The CPRD primary care database is, therefore, a rich source of health data for research, including data on demographics, symptoms, tests, diagnoses, therapies, health-related behaviors and referrals to secondary care [31]. At the mid-year date of 2 July 2013, there were 4.4 million active (alive, currently registered) patients meeting quality criteria, approximately covering 6.9% of the UK population. Patients are broadly representative of the UK general population in terms of age, sex, and ethnicity [31].

### Outcomes & covariates

Read diagnosis codes were used to identify all AOM episodes caused by all known and unknown pathogens in primary care. AOM included suppurative AOM, suppurative OM and unspecified OM (see Supplemental Table A1). In addition, AOM episodes were classified as simple AOM when referring to a single episode, or categorized as recurrent AOM if a patient had (1) three or more episodes within a 6-month period or (2) four or more episodes within a 12-month period, with at least one episode in the preceding 6 months [32]. Simple AOM episodes were considered resolved at the earliest date of the last GP visit with an AOM diagnosis followed by a ≥ 14 days gap without a GP visit with an AOM diagnosis, or at the date of death [23, 33, 34]. For episodes in which there was only a single GP visit with a Read diagnosis code for AOM, a period of 14 days was considered after that GP visit to account for antibiotic treatment. The reference date for the start of the 14-day gap was the date of the GP visit + 14 days.

To characterize the population, the demographic factors included age group, sex, geographic region, urbanicity, social deprivation, ethnicity, and medical risk conditions. Risk medical conditions were defined based on the Green Book chapter Pneumococcal Disease [35], and were divided into: asplenia or dysfunction of the spleen, chronic respiratory disease, chronic heart disease, chronic kidney disease, chronic liver disease, diabetes, immunocompromising diseases, cerebrospinal fluid leak, and cochlear implant. Social deprivation was measured using the 2019 English Index of Multiple Deprivation score (IMD), a patient postcode-linked measure for patients in English practices [36], which includes seven domains of deprivation: Income, Employment, Education, Health, Crime, Barriers to Housing & Services, and Living Environment [37]. To prevent disclosure of patient location, data are presented as quintiles of the deprivation score, with quintile 1 representing the least deprived areas and quintile 5 the most deprived areas [36]. The Rural-Urban classification was updated in 2011, defining areas as rural if they fall outside of areas forming settlements with populations of at least 10,000 resident population [36, 38].

### Statistical analyses

The statistical analyses were performed using SAS version 9.4 (SAS Institute, Inc., Cary, North Carolina). All descriptive analyses were conducted for children aged 0–17 years, and separately by age groups (< 2, 2–4, and 5–17 years), sex, region, urbanicity, social deprivation, and ethnicity. Regression analyses were performed for children aged 0–17 years, and separately by age groups.

AOM IRs per 100,000 person-years (PY) were calculated as the number of AOM episodes in a calendar year divided by the total number of PY among children aged 0–17 years in the database for each calendar year and for the following PCV risk time periods: pre-PCV7 (2003–2005); post-PCV7 (2007–2009); early post-PCV13 (2011–2014); and late post-PCV13 (2015–2019). The PCV risk time periods did not include the years 2006 and 2010, as they were periods of PCV implementation and setup (2006 for PCV7 and 2010 for PCV13) in the UK. The 95% confidence intervals (95% CI) for the IRs were calculated assuming a Poisson distribution. IRs were estimated for overall AOM, simple AOM, and recurrent AOM in each study year and PCV risk time period.

Time trends were analyzed using interrupted time series (ITS) models, using the pre-PCV7 (2003–2005) period as a baseline period, before any PCV was introduced. Baseline IRs were compared with the rates in the subsequent PCV time periods post-PCV7 (2007–2009); early post-PCV13 (2011–2014); and late post-PCV13 (2015–2019). Separate models were fitted overall and by each age stratum for the incidence of AOM episodes. As a first step, an exploratory analysis was conducted to verify and account for the presence of seasonality by plotting monthly IRs during the follow-up [39]. Scatterplots were used to identify the underlying trend, seasonal patterns, and outliers of time series data points. Monthly rates models were estimated using generalized linear models (GLM) with Poisson distribution and the log as link function (with population in log patient-years per 100,000 as an offset for the denominator). Adjustments were made to control for seasonality using annual trends within periods using Fourier terms. Incidence rate ratios (IRRs) and 95% CIs for each of the three PCV periods’ IRs were reported and compared to the pre-PCV7 (2003–2005) IR, the reference period before any PCV was introduced in the UK.

## Results

The study population included 1,500,686 children aged 0–17 years, contributing a total of 7,435,373.4 PY at risk. The study flowchart is summarized in Supplement Fig. A1. The characteristics of the study population at the inclusion date are presented in Table 1, the median age was 4.0 years (interquartile range 0.0–11.0) and 48.3% were female. Almost half of the children (48.6%) were aged 5–17 years (*N* = 729,689). The majority of the children (87.6%) lived in urban areas. The most populated regions were London (15.4%), the North West (15.0%), and the South East Coast (14.8%). Social deprivation, measured by the IMD score, was almost evenly distributed in the study population. Most children (93.2% [*N* = 1,398,469]) had no history of any of the selected risk medical conditions. Chronic respiratory disease was the most common risk medical condition observed in 6.2% (92,424) of the children. Due to high missing ethnicity information (75.1% not stated/missing/inconsistent), IRs by ethnic group were not reported.

**Table 1:** Baseline characteristics of the study population (*n* = 1,500,686) from 2003 to 2019

	N	%^**a**^
**Population by year**		
2003	527,885	–
2004	579,904	–
2005	627,223	–
2006	668,922	–
2007	707,332	–
2008	745,655	–
2009	766,870	–
2010	790,398	–
2011	790,747	–
2012	784,472	–
2013	772,412	–
2014	699,879	–
2015	585,480	–
2016	430,976	–
2017	341,078	–
2018	282,628	–
2019	243,745	–
**Age (years)**		
Minimum, maximum	0.0, 17.0	–
Mean, standard deviation	5.7, 5.7	–
Median (lower quartile, upper quartile)	4.0 (0.0–11.0)	–
**Age group**		
< 2 years	555,367	37.0
2–4 years	215,630	14.4
5–17 years	729,689	48.6
**Sex**		
Male	775,231	51.7
Female	725,455	48.3
**Geographic region**		
North East	29,145	1.9
North West	224,670	15.0
Yorkshire & The Humber	52,502	3.5
East Midlands	45,708	3.0
West Midlands	173,151	11.5
East of England	154,621	10.3
South West	175,419	11.7
South Central	191,935	12.8
London	231,471	15.4
South East Coast	222,064	14.8
**Urbanicity**		
Urban	1,314,679	87.6
Rural	186,007	12.4
**Social deprivation (IMD Score)**		
Quintile 1 (least deprived)	322,501	21.5
Quintile 2	293,616	19.6
Quintile 3	297,302	19.8
Quintile 4	289,738	19.3
Quintile 5 (most deprived)	295,851	19.7
Missing	1678	0.1
**Ethnicity**		
White	270,010	18.0
South Asian	43,354	2.9
Black	27,871	1.9
Other	14,403	1.0
Mixed	18,183	1.2
Not stated / Missing / Inconsistent	1,126,865	75.1
**Risk medical condition**		
No history of any risk medical condition	1,398,469	93.2
History of any risk medical condition^b^	102,217	6.8
Asplenia or dysfunction of the spleen	810	0.1
Chronic respiratory disease	92,424	6.2
Chronic heart disease	3806	0.3
Chronic kidney disease	585	0.0
Chronic liver disease	162	0.0
Diabetes	1946	0.1
Immunocompromising diseases	10,579	0.7
Cerebrospinal fluid leak	38	0.0
Cochlear implant	134	0.0

*Abbreviations*: *IMD* Index of Multiple Deprivation, *N* Number

^a^ Some totals sum to more or less than 100% due to rounding

^b^ The sum of each of the risk medical conditions does not match the total number of any risk medical condition because a patient could have more than one medical condition

### Incidence of AOM

**Fig. 1:**
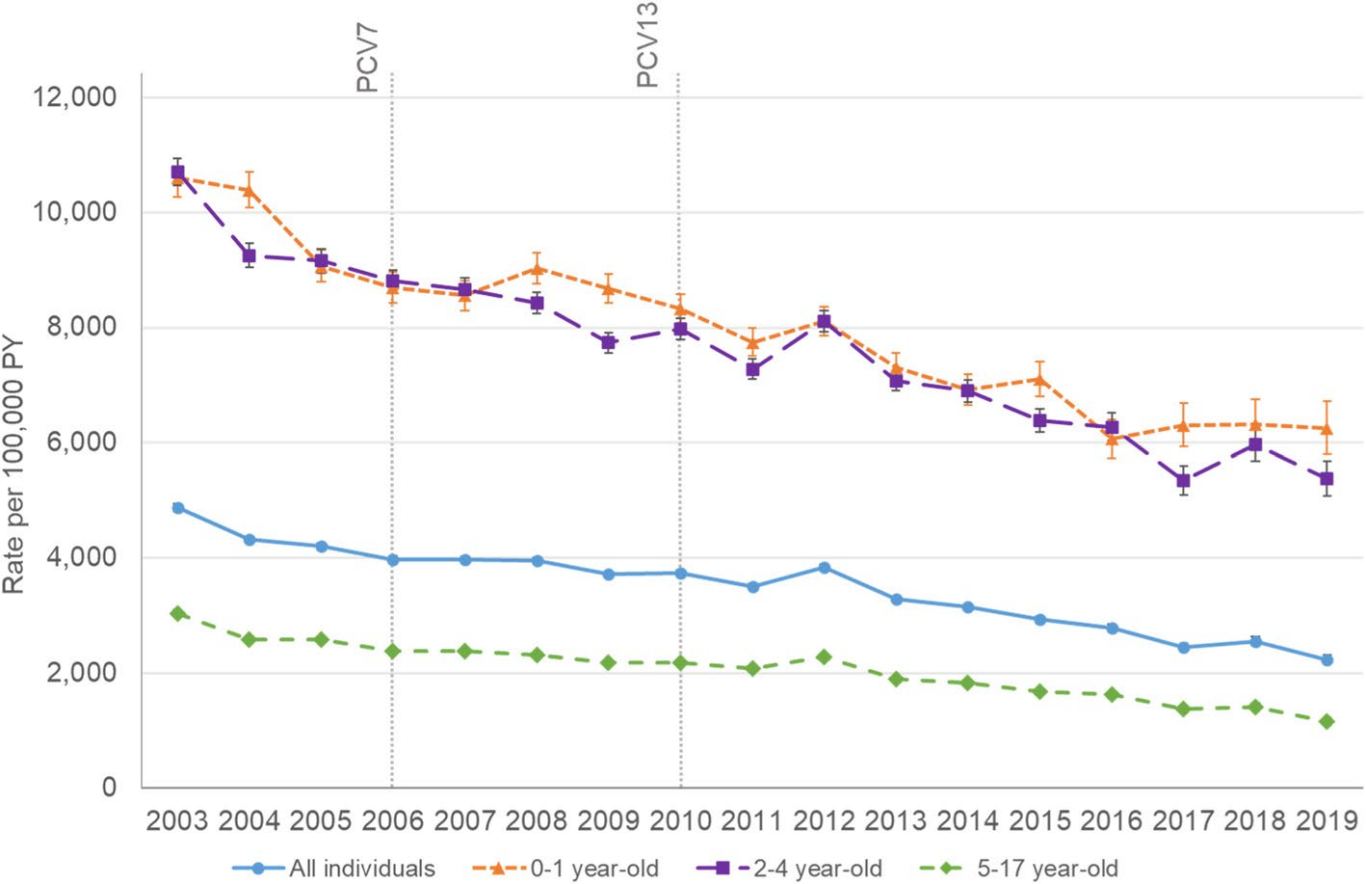
AOM IRs (per 100,000 PY) by year and by age groups (2003–2019). Abbreviations: AOM: acute otitis media; IRs: incidence rates; PCV: pneumococcal conjugate vaccine; PY: person-years

From 2003 to 2019, 274,008 episodes of AOM were identified in 1,500,686 children aged 0–17 years. The overall IR was 3690.9 (95% CI 3677.1-3704.8) per 100,000 PY. The overall IR of AOM per 100,000 PY was highest in children aged < 5 years and were as follows: 0 to 1 years: 8286.7 (95% CI 8216.8-8357.1); 2–4 years: 7951.8 (95% CI 7902.5-8001.4); 5–17 years: 2184.4 (95% CI 2172.1–2196.8) (Fig. 1 and Table 2). Overall IRs declined by 54.2% between 2003 and 2019 from 4871.4 (95% CI 4808.5-4934.9) to 2232.4 (95% CI 2156.7-2310.0) per 100,000 PY (Fig. 1 and Supplemental Table A2).

**Table 2:** AOM IRs by patient characteristics at the time of episode (2003–2019)

	N episodes	N PY at risk	Rate per 100,000 PY (95% CI)
**All individuals**	274,008	7,423,879.3	3690.9 (3677.1–3704.8)
**Age group**			
< 2 years	53,737	648,469.9	8286.7 (8216.8–8357.1)
2–4 years	99,641	1,253,057.0	7951.8 (7902.5–8001.4)
5–17 years	120,630	5,522,352.4	2184.4 (2172.1–2196.8)
**Sex**			
Male	144,196	3,839,363.1	3755.7 (3736.4–3775.2)
Female	129,812	3,584,516.3	3621.5 (3601.8–3641.2)
**Geographic region**			
North East	5380	156,221.8	3443.8 (3352.4–3537.1)
North West	47,586	1,221,914.1	3894.4 (3859.5–3929.5)
Yorkshire & The Humber	10,708	251,982.5	4249.5 (4169.4–4330.8)
East Midlands	7174	191,623.0	3743.8 (3657.7–3831.5)
West Midlands	39,151	904,505.1	4328.4 (4285.7–4371.5)
East of England	32,387	752,913.2	4301.6 (4254.8–4348.7)
South West	30,691	860,252.7	3567.7 (3527.9–3607.8)
South Central	40,621	988,238.4	4110.5 (4070.6–4150.6)
London	23,212	933,034.1	2487.8 (2455.9–2520.0)
South East Coast	37,098	1,163,194.4	3189.3 (3157.0–3221.9)
**Urbanicity**			
Urban	235,878	6,478,533.4	3640.9 (3626.2–3655.6)
Rural	38,130	945,345.9	4033.4 (3993.1–4074.1)
**Social deprivation (IMD Score)**			
Quintile 1 (least deprived)	69,264	1,770,303.3	3912.6 (3883.5–3941.8)
Quintile 2	57,076	1,492,482.6	3824.2 (3792.9–3855.7)
Quintile 3	55,562	1,458,851.2	3808.6 (3777.0–3840.4)
Quintile 4	49,036	1,352,620.8	3625.3 (3593.2–3657.5)
Quintile 5 (most deprived)	42,862	1,343,841.2	3189.5 (3159.4–3219.9)

*Abbreviations*: *AOM* Acute otitis media, *IMD* Index of Multiple Deprivation, *IR* Incidence rate, *N* Number, *PY* Person-years

Table 2 summarizes the IRs per 100,000 PY by patient characteristics at the time of episode. There were no differences in the IRs according to sex. Focusing on geographic region, the highest IRs were observed in children living in the West Midlands (4328.4 [95% CI 4285.7-4371.5]), and the lowest IRs were seen in London (2487.8 [95% CI 2455.9-2520.0]). Children living in rural areas had higher IRs than children living in urban areas, 4033.4 (95% CI 3993.1-4074.1) and 3640.9 (95% CI 3626.2-3655.6), respectively. Children living in the most affluent areas (quintile 1) had AOM IRs 23% higher compared to children living in the most deprived areas (quintile 5) (3912.6 [95% CI 3883.5-3941.8] versus 3189.5 [95% CI 3159.4-3219.9]).

### Incidence of simple versus recurrent AOM

Over the study period (2003–2019), 265,768 episodes of simple AOM were observed (IR 3579.7 [95% CI 3566.1-3593.4] per 100,000 PY). Children aged < 2 years had the highest IR overall: 7976.2 (95% CI 7907.6-8045.2) per 100,000 PY (Supplemental Table A3). The simple AOM IRs in the overall population almost halved (54.7%) across the study period: from 4791.1 (95% CI 4728.7-4854.0) in 2003 to 2171.2 (95% CI 2096.6, 2247.8) in 2019 (Supplemental Table A4).

The number of recurrent AOM episodes and IRs were lower compared to simple AOM, with 8240 recurrent AOM episodes identified and AOM recurrent IR of 110.8 (95% CI 108.5–113.3) per 100,000 PY. Children aged 2–4 years had the highest recurrent AOM IR overall: 332.3 per 100,000 PY (95% CI 322.3–342.5) (Supplemental Table A3). There was a decrease in recurrent AOM IRs across the study period, but this decline was less marked compared to simple AOM IRs. The recurrent AOM IRs in the overall population decreased by 23.6%, from 80.0 (95% CI 72.2–88.6) in 2003 to 61.1 (95% CI 49.1–75.0) in 2019, per 100,000 PY (Supplemental Table A5). Simple AOM and recurrent AOM IRs by patient characteristics (age, sex, geographic region, urbanicity and social deprivation) at the time of episode (2003–2019) are presented in Supplemental Table A3.

### AOM incidence by PCV periods

The overall AOM crude-incidence monthly ratios within period linear trends are presented in Supplemental Fig. A2, showing a decrease across the study period (2003–2019). AOM IRs declined by 40.3% overall between the pre-PCV7 period (2003–2005) and the late-PCV13 period (2015–2019) from 4451.9 (95% CI 4418.1-4485.9) to 2658.5 (95% CI 2628.6-2688.7), per 100,000 PY. By age group, a 34.9% reduction was observed for children aged < 2 years, from 9968.1 (95% CI 9792.9-10,145.6) to 6491.8 (95% CI 6329.1-6657.6) per 100,000 PY; a 38.2% decline for children aged 2–4 years, from 9684.5 (95% CI 9560.7-9809.6) to 5980.9 (95% CI 5871.6-6091.8); and a reduction by 45.1% in children 5–17 years, from 2729.0 (95% CI 2698.6-2759.7) to 1497.6 (95% CI 1471.7-1523.8) (Table 3).

**Table 3:** AOM IRs and AOM IRRs for IR change from the pre-PCV7 period, before and after the introduction of PCV7 and PCV13

	N episodes	Rate per 100,000 PY (95% CI)	IRR^**a**^ (95% CI) vs. reference period (pre-PCV7)^**b**^
**Pre-PCV7 (2003–2005)**			
Overall (all age-groups)	66,438	4451.9 (4418.1–4485.9)	1
<2 years	12,328	9968.1 (9792.9–10,145.6)	1
2–4 years	23,334	9684.5 (9560.7–9809.6)	1
5–17 years	30,776	2729.0 (2698.6–2759.7)	1
**Post-PCV7 (2007–2009)**			
Overall (all age-groups)	64,987	3879.2 (3849.4–3909.1)	0.87 (0.85–0.89)
< 2 years	13,259	8754.5 (8606.1–8904.8)	0.87 (0.82–0.93)
2–4 years	23,307	8267.4 (8161.6–8374.2)	0.80 (0.77–0.83)
5–17 years	28,421	2288.5 (2262.0–2315.2)	0.82 (0.79–0.85)
**Early Post-PCV13 (2011–2014)**			
Overall (all age-groups)	70,127	3462.8 (3437.2–3488.5)	0.88 (0.86–0.91)
< 2 years	13,833	7561.5 (7436.0–7688.6)	0.84 (0.79–0.89)
2–4 years	26,143	7365.2 (7276.2–7455.1)	0.87 (0.83–0.90)
5–17 years	30,151	2027.3 (2004.5–2050.3)	0.84 (0.81–0.87)
**Late Post-PCV13 (2015–2019)**			
Overall (all age-groups)	30,187	2658.5 (2628.6–2688.7)	0.75 (0.73–0.78)
< 2 years	6039	6491.8 (6329.1–6657.6)	0.72 (0.67–0.77)
2–4 years	11,388	5980.9 (5871.6–6091.8)	0.72 (0.69–0.75)
5–17 years	12,760	1497.6 (1471.7–1523.8)	0.68 (0.65–0.71)

*Abbreviations*: *AOM* Acute otitis media, *CI* Confidence interval, *IR* Incidence rate, *IRR* Incidence rate ratio, *N* Number, *PCV* Pneumococcal conjugate vaccine, *PY* Person-years

^a^ Adjusted for seasonal variations and within PCV period annual trend

^b^ Pre-PCV7 (2003–2005)

The IRR after adjusting for seasonal variations and the ‘within PCV period annual trend’ are also presented in Table 3. A significant decrease was observed in the overall population in the three post-vaccination periods: post-PCV7 (IRR 0.87, 95% CI 0.85–0.89, early post-PCV13 (IRR 0.88, 95% CI 0.86–0.91) and late post-PCV13 (IRR 0.75, 95% CI 0.73–0.78) compared to the pre-PCV7 reference period (2003–2005), when no PCV was introduced in the UK. A significant decrease was observed in the three age groups across the three post-PCV periods.

## Discussion

This study provides updated data on AOM incidence rates and trends since the introduction of PCVs in children in England. A better understanding of the AOM burden is important as new higher-valent pneumococcal conjugate vaccines are under development. Our findings indicate a 54.2% reduction of AOM incidence between 2003 (pre-PCV7 period) and 2019 (the late post-PCV13 period), and this decline was observed across all age groups. AOM IRs decreased with age, with highest IRs in children aged < 2 years and lowest in the eldest age group (5–17 years). Similar trends were observed for both simple and recurrent AOM IRs. IRRs indicated a significant decrease in AOM IRs in each of the post-vaccination periods, compared to the pre-PCV7 period before any PCV introduction in the UK. This significant decrease was also observed in the three age groups across the three post-PCV periods.

Our results are consistent with previous studies in England which have also reported declines in incidence of OM in the post-PCV periods. Lau WCY., et al. (2015) analyzed children aged < 10 years from the Integrated management system (IMS) Disease Analyser, a primary care database in the UK, and showed a 51.3% reduction of annual OM incidence between 2002 and 2012, from 135.8 episodes per 1000 PY in 2002 to 66.1 episodes per 1000 PY in 2012 [2]. In line with the results presented in this study, the introduction of PCV7 was associated with a significant decline in OM rates across all age-groups. Similarly, Thorrington D., et al. (2018), analyzed Hospital Episode Statistics (HES) data and showed a decline (IRR 0.78 (95% CI 0.76–0.81)) in the incidence of OM with tympanostomy among children under 15 years old between the pre-PCV era (1 April 2004–31 March 2006) and post-PCV era (1 April 2013–31 March 2015) [25]. Our results showed similar decline in IRs across PCV periods, although our incidence estimates are slightly different from those reported in Lau WCY., et al. and Thorrington D., et al. Several reasons may have influenced the different IR estimates: (1) the studies were conducted in different time periods; (2) the disease definitions studied differed (both Lau WCY., et al. and Thorrington D., et al. reported IRs for OM instead of AOM); (3) distinct data sources were used (Lau WCY., et al. used the IMS Disease Analyser database, including primary care data from the UK, and Thorrington D., et al. including all hospital admissions data in England); and (4) the average age of the study population was different. Furthermore, while the 14-days screening period between episodes was similar in Lau WCY., et al., the screening period was longer in Thorrington D., et al. (90 days), as the unit of analysis was hospital admissions [2, 25].

Other studies, elsewhere, have also observed significant declines in AOM incidence in the post-PCV era. A recent study in Israel demonstrated a downward trend in AOM IRs during the post-PCV years in children aged < 9 years (August 2009–2018; *p*-value < 0.001) with the largest decrease observed in children aged < 1 year [40]. Similar decreasing trends have also been observed in Sweden from 2005 to 2013 in children aged ≤5 years: with a 2.3% decrease in OM and AOM following PCV13 introduction [3]. In addition, a new study in Europe using a German health claims database (InGef - Institute for Applied Health Research Berlin, formerly Health Risk Institute) in children aged < 16 years from 2014 to 2019 reported significant declines in AOM IRs in all age groups over the study period [23]. In our study, the largest rate reduction by age group was seen in the 5–17 year-old age group (61.9%) between 2003 (pre-PCV7 period) and 2019 (the late post-PCV13 period).

In our study we found the incidence of AOM to be highest in the < 2 years age group compared to children aged 2–4 and 5–17 years. This is consistent with the Israeli and Swedish studies mentioned above [3, 40]. In Israel, children aged 0–1 and 1–2 years had the highest AOM episode rates in the pre-PCV years and the post-PCV years compared to children aged > 2 and < 9 years [40]. In Sweden, children aged ≤2 years had the highest IRs across all the study years compared to older children [3]. In contrast, in the German study children aged 2–4 years had the highest IRs [23].

Our results showed a 23.6% decrease in recurrent AOM IRs across the study period, but the decline was less marked compared to the 54.9% reduction in simple AOM IRs. To our knowledge, no other study conducted in the UK has previously reported simple and recurrent AOM IRs. Thorrington D., et al., reported a reduction of recurrent AOM requiring tympanostomy of 47% (95% CI 44–50%) and defined recurrent AOM as OM cases with procedure codes relating to the insertion, removal or maintenance of ventilation tubes through the tympanic membrane [25]. As the case definition used in Thorrington D., et al. differed from the one used in our study, it is not possible to compare the recurrent AOM IRs. In agreement with our results, the German study mentioned previously used similar case definitions for simple and recurrent AOM, and reported significant declines over the study period for both simple and recurrent AOM [23].

In our study, we observed that children from the most affluent areas (least deprived areas, Quintile 1), had 23% higher IRs compared to children living in the most deprived areas (Quintile 5). This is in line with a previous study conducted by the Royal College of General Practitioners (RCGP) Research and Surveillance Centre (RSC) in England that reported a higher likelihood of AOM among the least deprived compared to the most deprived [41]. This finding is likely explained by differences in health-seeking behavior across different socioeconomic groups. Previous reports have suggested that caregivers of infants from lower socioeconomic status are less likely to use healthcare services than caregivers from higher socioeconomic status [42]. A previous study reported that 67.7% of the children aged < 2 years with AOM had ear-related symptoms, concluding that AOM did not cause any specific symptoms that parents could use in their decision to seek medical advice for their child [43]. Further research is needed, however, to explore the relationship between the level of deprivation and AOM incidence.

A systematic review and appraisal of European National Clinical practice guidelines for AOM in children, including the UK, concluded that 15 of 17 (88%) of the European guidelines recommended a watchful waiting approach where clinicians were encouraged to prescribe antibiotics if symptoms persisted for 1–3 days or in case of any clinical deterioration [44]. The committee from the NICE Guideline for Otitis media (acute) agreed, based on the evidence, that no antibiotic prescription or a back-up antibiotic prescription could be considered for most children with AOM [13]. This watchful waiting approach may have influenced the health seeking behavior of parents for their child and therefore the true AOM incidence in the community is likely to be higher.

The main strength of this study is the size of the study population and representativeness of the CPRD-Gold database for the UK general population [31]. Another strength of our study is its design, excluding the years of implementation of the PCV7 and PCV13, 2006 and 2010 respectively, in our assessment of IR trends across PCV periods. This exclusion of the transition years between vaccination programs allows for a better estimation of the impact. This approach has been used previously in other studies [45, 46]. Another added value of this study was the inclusion of more years in the post-PCV13 period, and its division into early and late post-PCV13 periods, allowing for the observation of consistency of the effect of vaccination. It also allowed for the comparison of the effect in the short term with other studies. Previous studies conducted in the UK, have only included data up to 2012 (Lau WCY., et al. [2]) and 2015 (Thorrington D., et al. [25]).

A further strength lies in the choice of analysis method. When data are available for multiple time points in both the pre-intervention and the post-intervention periods, the ITS design offers a robust quasi-experimental alternative for evaluating treatment effects. The strength of ITS includes the ability to control for secular trends and seasonality in the data and the ability to evaluate outcomes using population-level data.

There were, however, a number of limitations to this study. First, there was the decrease in the number of patients in the study sample from 2015 onwards. This is explained by the migration of GP practices from Vision® software feeding into CPRD-Gold to EMIS software feeding into CPRD Aurum [47]. Despite this reduction in the size of the study population, CPRD-Gold is considered to be representative of the UK population [31]. Second, due to the requirement of a minimum of 6 months look-back period for the estimation of morbidity in children less than 12 months old, the true perinatal morbidity of infants is likely an underestimate. Similarly, the requirement for look-forward periods affected the study of recurrent AOM and limited the follow-up by 1 year, thereby likely underestimating the true IRs of recurrent AOM. Moreover, as the aim of our study was to estimate the overall, simple and recurrent AOM incidence rates, no patient-level adjustments at the time of the episode for being vaccinated or other covariates were included in our ITS models. Thus, IRs were presented as crude rates.

Finally, laboratory results and medical charts were not available to verify coding or diagnoses. This may have led to misclassification bias which may have overestimated the true incidence of AOM, nevertheless previous studies have used primary care data to identify AOM episodes in the UK [2]. All primary care providers who care for children will commonly care for those with AOM [48]. As GPs are the gatekeepers of care in the UK National Health Service [31], it is likely that a high number of AOM episodes have been captured. A referral to an otolaryngologist is only considered, among other reasons, if recurrent AOM, suspected complication of AOM, poor response to therapy, or associated hearing difficulties persist or progress [49].

Information on viral or bacterial AOM, causative pathogen (e.g., *Streptococcus pneumoniae, Haemophilus influenzae, Moraxella catarrhalis*) and serotype distribution were not available in the CPRD database. When considering the development and introduction of novel vaccines further research is needed to understand the prevalent and emerging pneumococcal serotypes to reduce residual disease burden, as well as understanding the adherence to the PCV vaccination schedule in England. In fact, the impact of novel higher-valent pneumococcal vaccines on AOM incidence rates will also depend on the proportion of AOM caused by circulating pneumococcal serotypes.

## Conclusions

This study presents recent estimates of AOM clinical burden in England. AOM incidence declined over the study period (2003–2019) in children aged ≤17 years in England following the introduction of PCV7 and PCV13. Despite the decrease in AOM incidence rates observed, the clinical burden of AOM remains substantial in children in England and, consequently, any associated healthcare resource use (e.g., primary care visits, antibiotic prescribing) and costs.

## Supplementary Information

**Additional file 1: Supplemental Table A1.** AOM Read diagnosis code definitions. **Supplement Fig. A1.** Study population flowchart. **Supplemental Table A2.** Overall AOM IRs by age group (2003–2019). **Supplemental Table A3.** Simple AOM and recurrent AOM IRs by patient characteristics at the time of episode (2003–2019). **Supplemental Table A4.** Simple AOM IRs by age group (2003–2019). **Supplemental Table A5.** Recurrent AOM IRs by age group (2003–2019). **Supplement Fig. A2.** Incidence monthly Ratio of AOM from 2003 to 2019.

## Data Availability

The data that support the findings of this study are available from CPRD, but restrictions apply to the availability of these data, which were used under license for the current study, and so are not publicly available. Data are however available from the authors upon reasonable request and with permission of CPRD. Please contact enquiries@cprd.com for further information.

## Abbreviations

AOM: Acute otitis media
CI: Confidence interval
CORE: Center for Observational and Real-World Evidence
CPRD: Clinical Practice Research Datalink
CSOM: Chronic suppurative otitis media
GLM: Generalized linear models
GP: General Practitioner
HES: Hospital Episode Statistics
IMD: Index of Multiple Deprivation
IMS: Integrated management system
IR: Incidence rate
IRR: Incidence rate ratio
ISAC: Independent Scientific Advisory Committee
ITS: Interrupted time series
JCVI: Joint Committee on Vaccination and Immunisation
NTHi: Non-typeable *Haemophilus influenzae*
NICE: National Institute for Health and Care Excellence
OM: Otitis media
OME: Otitis media with effusion
PCV: Pneumococcal conjugate vaccine
PCV13: 13-valent pneumococcal conjugate vaccine
PCV15: 15-valent pneumococcal conjugate vaccine
PCV20: 20-valent pneumococcal conjugate vaccine
PCV7: 7-valent pneumococcal conjugate vaccine
PY: Person-years
RCGP: Royal College of General Practitioners
RDAI: Real-world Data Analytics and Innovation
RSC: Research and Surveillance Centre
*S. pneumoniae*: *Streptococcus pneumoniae*
UK: United Kingdom
UTS: Up-to-standard
VAD: Value, Access and Devolved nations
VE: Vaccine effectiveness
