# Supplementary material for: Incidence of acute otitis media from 2003 to 2019 in children ≤ 17 years in England
Source: BMC Public Health. 2023 Jan 30;23:201. doi: 10.1186/s12889-023-14982-8 (PMC9885604; doi:10.1186/s12889-023-14982-8)
Supplement: Supplementary file 1 — Additional file 1: Supplemental Table A1. AOM Read diagnosis code definitions. Supplement Fig. A1. Study population flowchart. Supplemental Table A2. Overall AOM IRs by age group (2003–2019). Supplemental Table A3. Simple AOM and recurrent AOM IRs by patient characteristics at the time of episode (2003–2019). Supplemental Table A4. Simple AOM IRs by age group (2003–2019). Supplemental Table A5. Recurrent AOM IRs by age group (2003–2019). Supplement Fig. A2. Incidence monthly Ratio of AOM from 2003 to 2019. [file 12889_2023_14982_MOESM1_ESM.docx]

# Supplemental Appendix

**Supplemental table A1** AOM Read diagnosis code definitions

| **Read code** | **Read term** |
| --- | --- |
| F526.00 | Acute left otitis media |
| F528.00 | Acute bilateral otitis media |
| F527.00 | Acute right otitis media |
| F525.00 | Recurrent acute otitis media |
| SN30.11 | Aero-otitis media |
| A552.00 | Postmeasles otitis media |
| F520100 | Acute suppurative otitis media tympanic membrane ruptured |
| F520z00 | Acute suppurative otitis media NOS |
| F520.00 | Acute suppurative otitis media |
| F520300 | Acute suppurative otitis media due to disease EC |
| F520000 | Acute suppurative otitis media tympanic membrane intact |
| F52..00 | Suppurative and unspecified otitis media |
| F524000 | Bilateral suppurative otitis media |
| F523.00 | Chronic suppurative otitis media NOS |
| FyuP200 | [X]Other chronic suppurative otitis media |
| F52z.00 | Otitis media NOS |
| FyuP400 | [X]Otitis media in viral diseases classified elsewhere |
| FyuP300 | [X]Otitis media in bacterial diseases classified elsewhere |
| F521.00 | Chronic suppurative otitis media, tubotympanic |
| F522.00 | Chronic suppurative otitis media, atticoantral |

Abbreviations: AOM: acute otitis media; EC: elsewhere classified; NOS: not otherwise specified.

**Supplement Figure A1** Study population flowchart


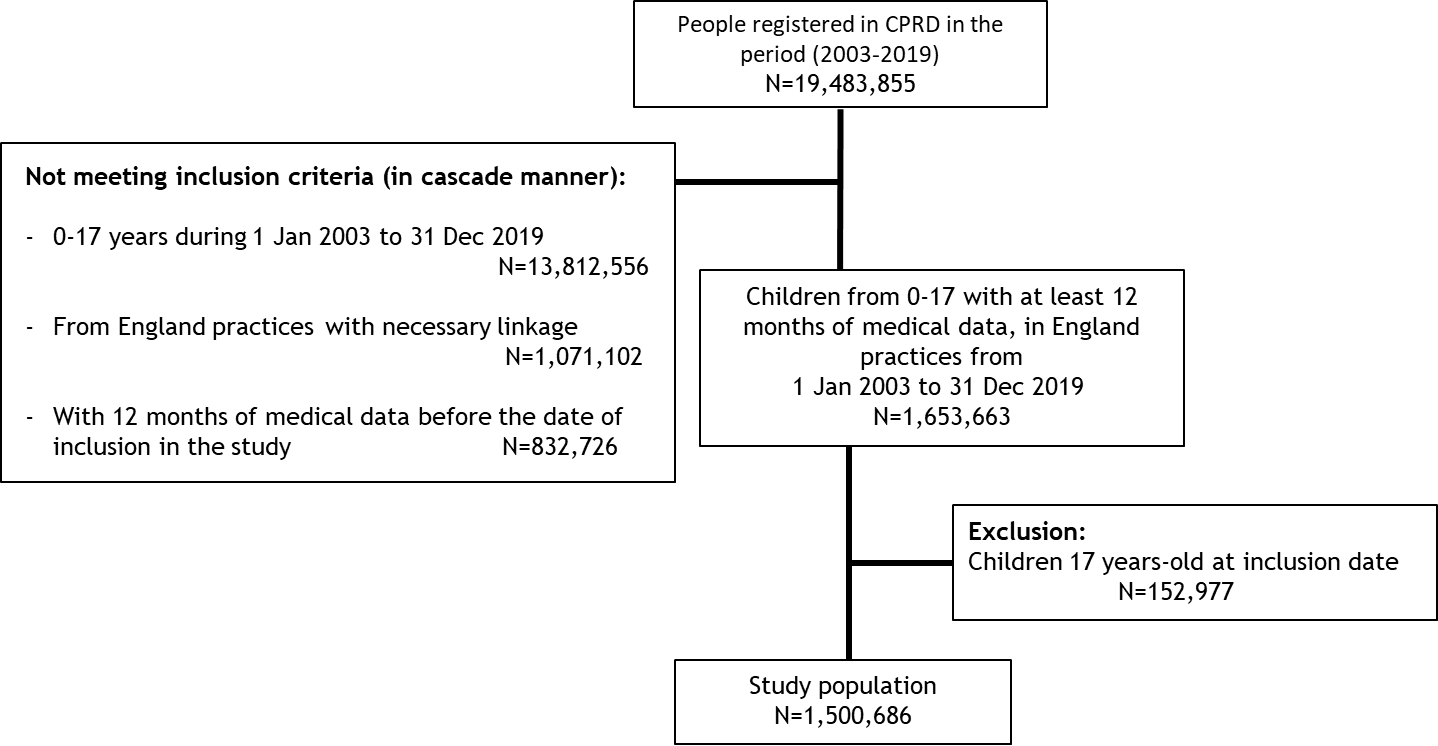


**Supplemental table A2** Overall AOM IRs by age group (2003-2019)

|  | **Overall** | | **<2 years** | | **2-4 years** | | **5-17 years** | |
| --- | --- | --- | --- | --- | --- | --- | --- | --- |
|  | **N episodes** | **Rate per 100,000 PY (95% CI)** | **N episodes** | **Rate per 100,000 PY (95% CI)** | **N episodes** | **Rate per 100,000 PY (95% CI)** | **N episodes** | **Rate per 100,000 PY (95% CI)** |
| **2003** | 22899 | 4871.4  (4808.5-4934.9) | 3799 | 10598.7  (10264.3-10941.2) | 8235 | 10704.5  (10474.6-10938.3) | 10865 | 3040.9  (2984.0-3098.6) |
| **2004** | 21629 | 4318.5  (4261.1-4376.4) | 4425 | 10390.3  (10086.4-10701.1) | 7425 | 9256.2  (9046.9-9469.2) | 9779 | 2586.7  (2535.7-2638.5) |
| **2005** | 21910 | 4201.8  (4146.4-4257.8) | 4104 | 9070.9  (8795.5-9352.8) | 7674 | 9158.1  (8954.4-9365.4) | 10132 | 2582.0  (2532.0-2632.8) |
| **2006** | 21375 | 3978.1  (3925.0-4031.8) | 4059 | 8687.4  (8422.2-8958.9) | 7713 | 8806.7  (8611.3-9005.5) | 9603 | 2382.8  (2335.4-2431.0) |
| **2007** | 21813 | 3967.4  (3914.9-4020.4) | 4211 | 8553.7  (8297.3-8816.0) | 7882 | 8666.0  (8475.7-8859.5) | 9720 | 2372.9  (2325.9-2420.5) |
| **2008** | 22175 | 3952.8  (3901.0-4005.2) | 4605 | 9026.5  (8767.7-9291.0) | 7966 | 8424.4  (8240.4-8611.4) | 9604 | 2311.9  (2265.9-2358.6) |
| **2009** | 20999 | 3720.1  (3669.9-3770.7) | 4443 | 8676.6  (8423.3-8935.6) | 7459 | 7737.3  (7562.7-7914.9) | 9097 | 2182.2  (2137.6-2227.5) |
| **2010** | 20894 | 3742.4  (3691. 9-3793.5) | 4219 | 8329.2  (8079.8-8584.4) | 7756 | 7974.3  (7797.8-8153.7) | 8919 | 2173.3  (2128.5-2218.9) |
| **2011** | 19098 | 3505.2  (3455.7-3555.3) | 3891 | 7739.7  (7498.4-7986.8) | 6942 | 7275.8  (7105.6-7449.0) | 8265 | 2070.6  (2026.2-2115.78) |
| **2012** | 20639 | 3836.1  (3784.0-3888.8) | 4019 | 8101.2  (7852.7-8355.6) | 7677 | 8105.9  (7925.5-8289.2) | 8943 | 2271.5  (2224.7-2319.1) |
| **2013** | 16626 | 3286.2  (3236.4-3336.5) | 3333 | 7305.9  (7059.9-7558.2) | 6263 | 7074.3  (6900.2-7251.8) | 7030 | 1890.9  (1846.9-1935.6) |
| **2014** | 13764 | 3154.4  (3101.9-3207.5) | 2590 | 6918.4  (6654.5-7190.1) | 5261 | 6895.2  (6710.2-7084.1) | 5913 | 1832.8  (1786.4-1880.2) |
| **2015** | 10315 | 2930.0  (2873.7-2987.1) | 2079 | 7097.7  (6795.8-7409.5) | 3857 | 6381.4  (6181.5-6586.0) | 4379 | 1669.4  (1620.3-1719.6) |
| **2016** | 7113 | 2780.7  (2716.4-2846.1) | 1289 | 6059.2  (5732.9-6399.2) | 2724 | 6273.6  (6040.2-6513.7) | 3100 | 1622.1  (1565.5-1680.2) |
| **2017** | 5026 | 2440.4  (2373.4-2508.8) | 1092 | 6299.8  (5931.7-6684.8) | 1821 | 5340.0  (5097.6-5591.1) | 2113 | 1367.5  (1309.8-1427.1) |
| **2018** | 4445 | 2548.9  (2474.5-2625.0) | 869 | 6314.7  (5901.8-6748.9) | 1709 | 5961.6  (5682.3-6251.1) | 1867 | 1414.8  (1351.4-1480.5) |
| **2019** | 3288 | 2232.4  (2156.7-2310.0) | 710 | 6247.2  (5796.0-6724.1) | 1277 | 5371.1  (5080.5-5674.0) | 1301 | 1160.1  (1097.9-1224.9) |

Abbreviations: AOM: acute otitis media; CI: confidence interval; IR: incidence rate; N: number; PY: person-years.

**Supplemental table A3** Simple AOM and recurrent AOM IRs by patient characteristics at the time of episode (2003-2019)

|  | **Simple AOM** | | | **Recurrent AOM** | | |
| --- | --- | --- | --- | --- | --- | --- |
|  | **N episodes** | **N PY at risk** | **Rate per 100,000 PY**  **(95% CI)** | **N episodes** | **N PY at risk** | **Rate per 100,000 PY**  **(95% CI)** |
| **All individuals** | 265768 | 7424238.8 | 3579.7 (3566.1, 3593.4) | 8240 | 7435014.5 | 110.8 (108.5, 113.3) |
| **Age group** |  |  |  |  |  |  |
| <2 years | 51730 | 648557.1 | 7976.2 (7,907.6, 8,045.2) | 2007 | 650601.0 | 308.5 (295.1, 322.3) |
| 2-4 years | 95464 | 1253240.2 | 7617.4 (7,569.1, 7,665.9) | 4177 | 1257081.3 | 332.3 (322.3, 342.5) |
| 5-17 years | 118574 | 5522441.5 | 2147.1 (2,134.9, 2,159.4) | 2056 | 5527332.3 | 37.2 (35.6, 38.8) |
| **Sex** |  |  |  |  |  |  |
| Male | 139450 | 3839570.6 | 3631.9 (3612.9, 3651.0) | 4746 | 3845210.8 | 123.4 (119.9, 127.0) |
| Female | 126318 | 3584668.2 | 3523.8 (3504.4, 3543.3) | 3494 | 3589803.7 | 97.3 (94.1, 100.6) |
| **Geographic region** |  |  |  |  |  |  |
| North East | 5221 | 156229.0 | 3341.9 (3251.9, 3433.8) | 159 | 156441.7 | 101.6 (86.5, 118.7) |
| North West | 46063 | 1221980.2 | 3769.5 (3735.2, 3804.1) | 1523 | 1223846.1 | 124.4 (118.3, 130.9) |
| Yorkshire & The Humber | 10378 | 251996.6 | 4118.3 (4039.5, 4198.3) | 330 | 252417.5 | 130.7 (117.0, 145.6) |
| East Midlands | 6931 | 191633.4 | 3616.8 (3532.2, 3703.0) | 243 | 191914.8 | 126.6 (111.2, 143.6) |
| West Midlands | 37792 | 904565.5 | 4177.9 (4135.9, 4220.3) | 1359 | 906093.3 | 150.0 (142.1, 158.2) |
| East of England | 31396 | 752956.4 | 4169.7 (4123.7, 4216.1) | 991 | 754226.5 | 131.4 (123.3, 139.8) |
| South West | 29837 | 860289.7 | 3468.3 (3429.0, 3507.8) | 854 | 861503.5 | 99.1 (92.60, 106.0) |
| South Central | 39326 | 988294.6 | 3979.2 (3940.0, 4018.7) | 1295 | 989886.5 | 130.8 (123.8, 138.2) |
| London | 22692 | 933056.5 | 2432.0 (2400.5, 2463.9) | 520 | 933981.7 | 55.7 (51.0, 60.7) |
| South East Coast | 36132 | 1163237.0 | 3106.2 (3074.2, 3138.4) | 966 | 1164703.0 | 82.9 (77.8, 88.3) |
| **Urbanicity** |  |  |  |  |  |  |
| Urban | 228863 | 6478839.3 | 3532.5 (3518.0, 3547.0) | 7015 | 6488119.6 | 108.1 (105.6, 110.7) |
| Rural | 36905 | 945399.4 | 3903.6 (3863.9, 3943.7) | 1225 | 946895.0 | 129.4 (122.2, 136.8) |
| **Social deprivation (IMD Score)** |  |  |  |  |  |  |
| Quintile 1 (least deprived) | 67137 | 1770396.3 | 3792.2 (3763.6, 3821.0) | 2127 | 1773119.6 | 120.0 (114.9, 125.2) |
| Quintile 2 | 55249 | 1492562.8 | 3701.6 (3670.8, 3732.6) | 1827 | 1494803.0 | 122.2 (116.7, 128.0) |
| Quintile 3 | 53907 | 1458923.0 | 3695.0 (3663.9, 3726.3) | 1655 | 1461109.0 | 113.3 (107.9, 118.9) |
| Quintile 4 | 47509 | 1352687.3 | 3512.2 (3480.7, 3543.9) | 1527 | 1354609.8 | 112.7 (107.1, 118.5) |
| Quintile 5 (most deprived) | 41760 | 1343889.1 | 3107.4 (3077.7, 3137.4) | 1102 | 1345584.3 | 81.9 (77.1, 86.9) |

Abbreviations: AOM: acute otitis media; IMD: Index of Multiple Deprivation; IR: incidence rate; N: number; PY: person-years.

**Supplemental table A4** Simple AOM IRs by age group (2003-2019)

|  | **Overall** | | **<2 years** | | **2-4 years** | | **5-17 years** | |
| --- | --- | --- | --- | --- | --- | --- | --- | --- |
|  | **N episodes** | **Rate per 100,000 PY (95% CI)** | **N episodes** | **Rate per 100,000 PY (95% CI)** | **N episodes** | **Rate per 100,000 PY (95% CI)** | **N episodes** | **Rate per 100,000 PY (95% CI)** |
| **2003** | 22522 | 4791.1  (4728.7-4854.0) | 3692 | 10298.9  (9969.4-10636.6) | 8054 | 10468.2  (10240.8-10699.4) | 10776 | 3016.0  (2959.3-3073.5) |
| **2004** | 20775 | 4147.7  (4091.4-4204.4) | 4229 | 9928.1  (9631.1-10231.9) | 7012 | 8739.4  (8536.0-8946.4) | 9534 | 2521.9  (2471.5-2573.0) |
| **2005** | 21131 | 4052.2  (3997.7-4107.2) | 3923 | 8669.3  (8400.1-8944.9) | 7286 | 8693.3  (8494.8-8895.3) | 9922 | 2528.5  (2479.0-2578.7) |
| **2006** | 20667 | 3846.2  (3793.9-3899.0) | 3906 | 8358.7  (8098.6-8625.1) | 7356 | 8397.6  (8206.8-8591.7) | 9405 | 2333.7  (2286.7-2381.3) |
| **2007** | 21123 | 3841.7  (3790.0-3893.8) | 4041 | 8207.1  (7956.0-8464.1) | 7531 | 8278.7  (8092.8-8467.8) | 9551 | 2331.6  (2285.1-2378.8) |
| **2008** | 21471 | 3827.1  (3776.1-3878.7) | 4439 | 8699.9  (8445.9-8959.7) | 7610 | 8046.6  (7866.8-8229.4) | 9422 | 2268.0  (2222.5-2314.3) |
| **2009** | 20359 | 3606.5  (3557.1-3656.4) | 4266 | 8329.7  (8081.6-8583.5) | 7131 | 7396.0  (7225.3-7569.7) | 8962 | 2149.8  (2105.5-2194.8) |
| **2010** | 20328 | 3640.9  (3591.0-3691.3) | 4088 | 8069.7  (7824.2-8320.9) | 7425 | 7632.8  (7460.2-7808.4) | 8815 | 2148.0  (2103.4-2193.3) |
| **2011** | 18521 | 3399.2  (3350.4-3448.5) | 3767 | 7492.3  (7254.9-7735.4) | 6655 | 6974.0  (6807.5-7143.7) | 8099 | 2029.0  (1985.1-2073.7) |
| **2012** | 19988 | 3714.9  (3663.6-3766.8) | 3838 | 7735.2  (7492.4-7983.8) | 7362 | 7772.1  (7595.6-7951.7) | 8788 | 2232.1  (2185.7-2279.3) |
| **2013** | 16082 | 3178.5  (3129.6-3228.0) | 3207 | 7028.9  (6787.7-7276.5) | 5986 | 6760.5  (6590.3-6934.0) | 6889 | 1852.9  (1809.4-1897.2) |
| **2014** | 13369 | 3063.7  (3012.0-3116.1) | 2478 | 6618.3  (6360.3-6884.2) | 5084 | 6662.6  (6480.7-6848.3) | 5807 | 1800.0  (1754.0-1846.9) |
| **2015** | 10021 | 2846.4  (2790.9-2902.7) | 2002 | 6834.0  (6537.9-7140.1) | 3711 | 6139.1  (5943.2-6339.9) | 4308 | 1642.3  (1593.6-1692.1) |
| **2016** | 6932 | 2709.8  (2646.4-2774.4) | 1258 | 5913.1  (5590.8-6249.1) | 2610 | 6010.4  (5782.0-6245.5) | 3064 | 1603.3  (1547.0-1661.1) |
| **2017** | 4920 | 2388.8  (2322.6-2456.5) | 1066 | 6149.4  (5785.8-6530.0) | 1761 | 5163.7  (4925.3-5410.6) | 2093 | 1354.5  (1297.1-1413.8) |
| **2018** | 4361 | 2500.7  (2427.0-2576.0) | 853 | 6198.1  (5789.1-6628.4) | 1660 | 5790.2  (5515.0-6075.6) | 1848 | 1400.4  (1337.3-1465.8) |
| **2019** | 3198 | 2171.2  (2096.6-2247.8) | 677 | 5956.1  (5515.8-6422.1) | 1230 | 5173.0  (4887.9-5470.4) | 1291 | 1151.2  (1089.2-1215.7) |

Abbreviations: AOM: acute otitis media; CI: confidence interval; IR: incidence rate; N: number; PY: person-years.

**Supplemental table A5** Recurrent AOM IRs by age group (2003-2019)

|  | **Overall** | | **<2 years** | | **2-4 years** | | **5-17 years** | |
| --- | --- | --- | --- | --- | --- | --- | --- | --- |
|  | **N episodes** | **Rate per 100,000 PY (95% CI)** | **N episodes** | **Rate per 100,000 PY (95% CI)** | **N episodes** | **Rate per 100,000 PY (95% CI)** | **N episodes** | **Rate per 100,000 PY (95% CI)** |
| **2003** | 377 | 80.0 (72.2-88.6) | 107 | 297.3 (243.6-359.2) | 181 | 234.3 (201.4-271.0) | 89 | 24.9 (20.0-30.6) |
| **2004** | 854 | 170.2 (159.0-182.0) | 196 | 458.3 (396.4-527.2) | 413 | 513.0 (464.7-564.9) | 245 | 64.7 (56.9-73.4) |
| **2005** | 779 | 149.1 (138.9-160.0) | 181 | 398.6 (342.7-461.1) | 388 | 461.3 (416.6-509.6) | 210 | 53.5 (46.5-61.2) |
| **2006** | 708 | 131.6 (122.0-141.6) | 153 | 326.3 (276.7-382.3) | 357 | 406.2 (365.1-450.6) | 198 | 49.1 (42.5-56.4) |
| **2007** | 690 | 125.3 (116.1-135.0) | 170 | 344.2 (294.4-400.0) | 351 | 384.6 (345.4-427.0) | 169 | 41.2 (35.2-47.9) |
| **2008** | 704 | 125.3 (116.2-134.9) | 166 | 324.2 (276.8-377.5) | 356 | 375.2 (337.3-416.3) | 182 | 43.8 (37.6-50.6) |
| **2009** | 640 | 113.2 (104.6-122.3) | 177 | 344.5 (295.6-399.1) | 328 | 339.2 (303.5-377.9) | 135 | 32.4 (27.1-38.3) |
| **2010** | 566 | 101.2 (93.1-109.9) | 131 | 257.8 (215.5-305.9) | 331 | 339.2 (303.7-377.8) | 104 | 25.3 (20.7-30.7) |
| **2011** | 577 | 105.8 (97.3-114.7) | 124 | 245.9 (204.5-293.2) | 287 | 299.9 (266.2-336.7) | 166 | 41.6 (35.5-48.4) |
| **2012** | 651 | 120.8 (111.7-130.5) | 181 | 363.7 (312.6-420.7) | 315 | 331.5 (295.9-370.2) | 155 | 39.3 (33.4-46.0) |
| **2013** | 544 | 107.4 (98.5-116.8) | 126 | 275.4 (229.4-327. 9) | 277 | 312.0 (276.3-351.0) | 141 | 37.9 (31.9-44.7) |
| **2014** | 395 | 90.4 (81.7-99.8) | 112 | 298.4 (245.7-359.0) | 177 | 231.3 (198.5-268.0) | 106 | 32.8 (26.9-39.7) |
| **2015** | 294 | 83.4 (74.2-93.5) | 77 | 262.1 (206.9-327.6) | 146 | 240.9 (203.4-283.3) | 71 | 27.1 (21.1-34.1) |
| **2016** | 181 | 70.7 (60.8-81.8) | 31 | 145.4 (98.8-206.3) | 114 | 261.9 (216.0-314.6) | 36 | 18.8 (13.2-26.1) |
| **2017** | 106 | 51.4 (42.1-62.2) | 26 | 149.6 (97.7-219.2) | 60 | 175.6 (134.0-226.0) | 20 | 12.9 (7.9-20.0) |
| **2018** | 84 | 48.1 (38.4-59.6) | 16 | 116.0 (66.3-188.3) | 49 | 170.5 (126.2-225.4) | 19 | 14.4 (8.7-22.5) |
| **2019** | 90 | 61.1 (49.1-75.0) | 33 | 289.7 (199.4-406.8) | 47 | 197.3 (144.9-262.3) | 10 | 8.9 (4.3-16.4) |

Abbreviations: AOM: acute otitis media; CI: confidence interval; IR: incidence rate; N: number; PY: person-years.

**Supplement Figure A2** Incidence monthly Ratio of AOM from 2003 to 2019


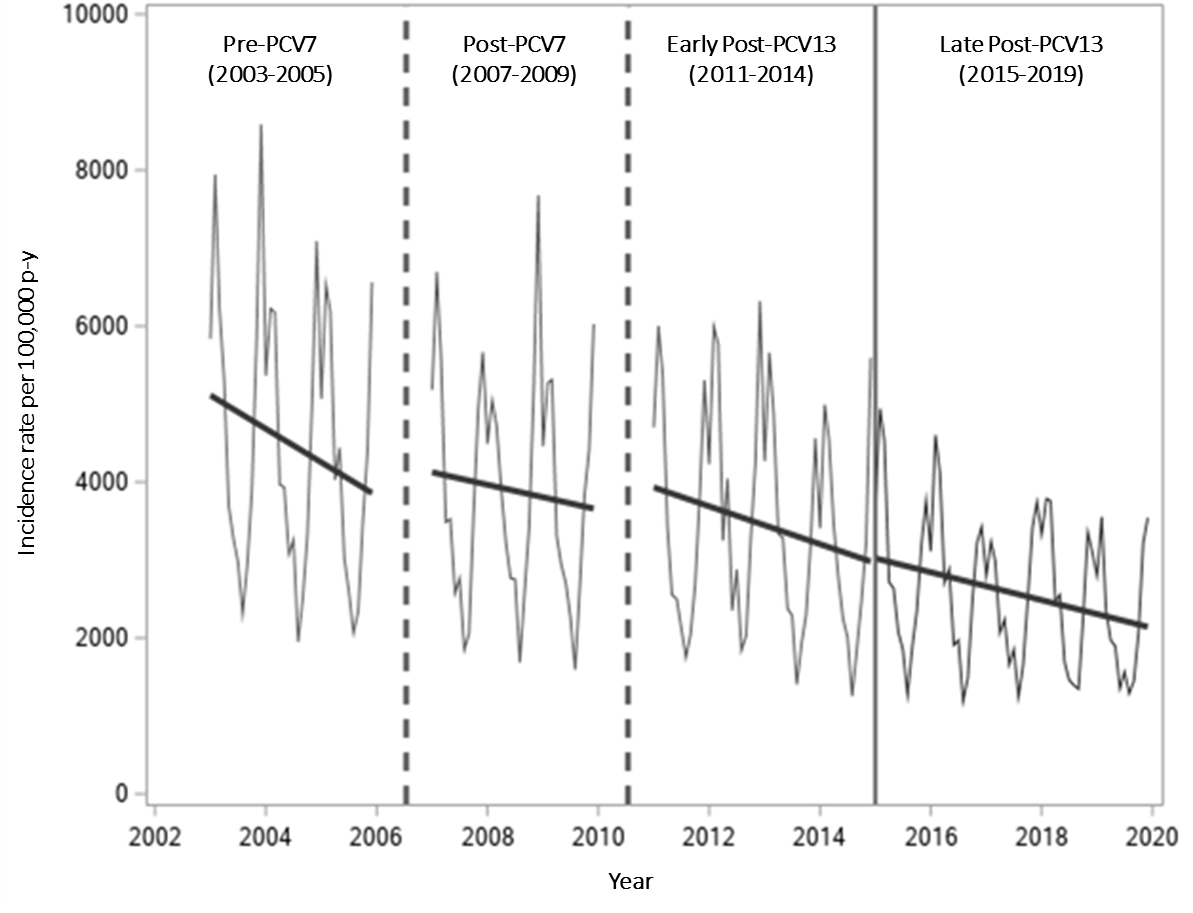


Abbreviations: AOM: acute otitis media; PCV: pneumococcal conjugate vaccine; PY: person-years. The dashed vertical lines correspond to the years of PCV introduction (2006 and 2010) where monthly episode rates of these years were not calculated. The solid vertical line denotes the separation of early and late PCV13 periods-where all years were included in the monthly episode rates.
